# The Treatment of Acute Rheumatism, Illustrated by Cases Taken from Private Practice

**Published:** 1850-10

**Authors:** Geo. L. Upshur

**Affiliations:** Norfolk, Va., Member of the American Medical Association


*The Treatment of Acute Rheumatism, illustrated by cases taken from private practice.* By Geo. L. Upshur, M.D., Norfolk, Va., Member of the American Medical Association.

Case 1.—Mrs. W., aged 30, of sanguineous temperament, and rather delicate, was seized with pain in the right shoulder joint on 23d of September, 1846. The weather had been inclement for several days previous, and she had imprudently exposed herself. I saw her on the 24th, at which time all the large joints were somewhat painful, although there was little or no fever.—*Prescription.* ℞. Pulv. Dover. gr. xv. Div. in chart. 3. S. one powder every three hours.

25th. Patient much more uncomfortable; slept none last night; nearly every joint red, swollen, and exquisitely tender; pulse, full, hard, and bounding; bowels confined; tongue foul, thirst urgent, and perspiration very profuse. Being desirous of avoiding, if possible, the use of the lancet in so delicate a subject, I ordered only the following: ℞. Vin. Colch. Rad. f.ʒiss.; Magnes. calc. ʒij.; Magnes. Sulph. ℥ss.; aquæ f.℥iv. M. S. A table-spoonful every three hours, until it purges freely. At 9 o'clock P. M. the mixture had produced several copious stools, but without relief to any of the symptoms; took 20 ounces of blood from the arm.

26th. Symptoms somewhat improved; less fulness of the pulse, much less perspiration, and not so much pain. ℞. Quiniæ Sulph. ʒss. Div. in chart. 3. S. One powder every three hours.

On the 27th, my patient was so entirely relieved, that I considered her convalescing, and ceased to visit her after the 28th.

Case 2.—Miss B., aged 19, very large and robust, was taken with chill followed by high fever, with metastatic pains in the joints and a profuse warm perspiration, on 14th of January 1847. Sent for me in the night, and I have never seen a better marked case of acute articular rheumatism—even the joints of the fingers and toes were red and swollen. The blood was fairly running riot through the vessels, and the patient alternately shrieked and ground her teeth in very agony. I bled her to full 25 ounces, which checked the perspiration, and lessened the pain in a considerable degree; ordered gr. x. of Dover's powder to be taken at once.

15th. Somewhat more comfortable; still suffering very much. ℞. Ol. Tiglii gtt. ij.; Saponis Hisp. q. s. M. Pil. 2. S. Give one pill immediately, and if it does not act in four hours, the other.

16th. Took both pills which acted copiously; passed a comparatively good night; and is decidedly better this morning; pills vomited her freely; ℞. Calomel gr. x. Ol.Tiglii. gtt. iss. M. Pil. 3. S. one pill every hour.

On the 17th the patient was on the verge of convalescence; ordered ten grains of Dover's powder at bed-time.

18th. Improving; opened the bowels with croton oil.

19th. Pain nearly subsided; no fever; appetite returning. Ordered a table-spoonful, three times a day, of the colchicum mixture, to keep the bowels soluble. On the 24th discharged her cured.

Case 3.—Mr. E., aged 22, sanguineous temperament, was seized with acute articular rheumatism, May 16th, 1847; saw him on 17th; pain confined chiefly to shoulders, elbows, and wrists, which were red and swollen. Prescribed croton oil in purgative doses followed by the colchicum, magnesia, &c. as in other cases. Discharged him convalescent on the 19th. On the the 6th of August following he was again attacked, and again relieved, in a great degree, by the croton oil. The disease, however, became chronic in the course of two or three weeks, when I gave him the vinum colchici, and subsequently the iodide of potassium with the fluid extract of sarsaparilla, with fine effect; I also derived great benefit from "firing" around the joints as recommended by Dr. Corrigan in the *Dublin Hospital Gazette* for March 1846. The article is too long to insert here, but it may be found, condensed, in vol. 4th of Ranking's Abstract, p. 102, and is well worth a perusal.

About four weeks after the disease assumed the chronic form, there appeared upon the extremities, a pustular eruption, resembling very closely, the eruption of small pox. The base of the pustules, however, instead of being red as in variola, was livid and variegated, putting one in mind of the blotches seen in secondary syphilis. I have no doubt but that there was a syphilitic taint in this case. After two or three months, Mr. E. returned to his friends in New England, much improved, but not entirely relieved. I have not heard whether he ever permanently recovered.

Case 4.—Mrs. Wiles, a very delicate lady, aged 25, mother of two children, and withal a confirmed dyspeptic, was taken sick with acute rheumatism in its severest form, May 29th, 1847. When I saw her on the 30th, her fever was high, the pulse frequent without fulness, perspiration profuse, bowels confined, and pain in the joints unbearable. Purged her violently with croton oil, and on the next day, 31st, gave ʒi. of Pulv. Jal. Comp., which acted well.

June 1st. Much better; fever, pain and perspiration greatly lessened; determined to give the colchicum a fair trial. I gave it to her without intermission, until the 8th, but apparently without benefit; I then prescribed quinine, ten grains three times a day; she took in all sixty grains, and was discharged cured on the 12th of June, having been ill just two weeks.

Case 5.—Miss M., aged 30, robust, was seized March 28th, 1848, with chill. When I saw her, all her joints were involved, and there was scarcely a voluntary muscle in the body that could be moved without pain; fever high, abundant perspiration, and constipated bowels; ℞. Ol. Tiglii gtt. vi.; Calomel gr. xxx. M. Pil. 6. ℞. Vin. Colch. R. f.ʒi.; Magnes. Calc. ʒij.; Magnes. Sulph. ʒiij.; Aqua f.℥iv. M. I directed her to take one of the pills immediately, and after it ceased to operate, to begin with the mixture and take a table-spoonful every three hours. On visiting her next morning, I was alarmed at her condition. She was excessively feeble, pallid, with her extremities cold and shrivelled, and passing blood from the bowels every ten minutes; each stool being accompanied with violent straining and tenesmus. The whole abdomen was tender upon pressure; with nausea, and a frequent, feeble pulse. *With all this, however, she had not even a remnant of the rheumatism, and could move her limbs about in any position without the slightest inconvenience.*

Upon inquiry, I ascertained that the nurse, a listless, inattentive creature, had mistaken my directions entirely, by giving the *pills* as I ordered her to give the *mixture*, namely, one pill every three hours. The patient took six drops of croton oil, and thirty grains of calomel, within eighteen hours, and, as a matter of course, was purged nearly to death. The only wonder is, that she did not die before I saw her. She recovered slowly from the effects of the hypercatharsis, and has not had a rheumatic pain from that day to this!

Case 6.—Frances B., aged 12, lymphatic temperament, seized Sept. 23d, 1848, with well marked acute articular rheumatism; the upper joints chiefly affected, but the disease showing a decided tendency towards metastasis. Prescribed Ol. Tiglii gtt. i.; Calomel gr. v. M. S. To be taken immediately. The following day there was a decided amelioration of all the symptoms. ℞. Quiniæ Sulph. gr. xxx; Div. in chart. 4. One powder to be given every two hours. The patient was well enough to sit up on the 26th, when I ceased to visit her.

Case 7.—On the 3d of October, 1848, I was called to see Mr. W., aged 46. He had been in feeble health for a year or two before, and was then suffering from a chronic ulcer upon the left leg. For several days he had been troubled with sharp rheumatic pains in the shoulders and back of the neck. He had very little fever; bowels confined. Gave him a table-spoonful of the colchicum mixture every three hours. The next day he was rather worse, when I purged him with croton oil. On the 6th, purged him again with the same, and again on the 8th, and on the 9th I found him entirely relieved. In a subsequent attack, I gave him the decoction of may-apple (Podophyllum) which acted copiously on the bowels, and gave him great relief.

Case 8.—Saw William S., aged 40, October 11th, 1848; fever, constipated bowels, and pain, redness and swelling of all the large joints. Prescribed Ol. Tiglii gtt. ij., to be taken at once; repeated the dose the following day, and on the 13th found him so much relieved that he required no further treatment.

Case 9.—Mr. Hubert S., an athletic young man of sanguineous temperament, was seized Nov. 28th, 1848. Three years before, he had an attack of rheumatism, which confined him to the bed four weeks. When I saw him, the ankle and knee joints only were very painful, with now and then a twinge in the shoulders. He had but little fever, and no perspiration. Prescribed the croton oil as in the other cases, and repeated the dose on the next day. The symptoms were relieved entirely, with the exception of slight pain and stiffness in the right ankle joint. Ordered 5 grs. of iodide of potassium, to be taken four times a day in infusion of hops; under this treatment he recovered in about ten days.

Case 10.—Mr. John M., was taken violently on the 8th of December, 1848. He was a fleshy, muscular man, and suffered intensely. Gave him the croton oil as usual, followed by eight grains of quinine every hour, until he had taken 40 grains. He was entirely relieved on the 10th, and had no return of the disease.

Case 11.—Mrs. F., aged 26, recently marrried, anemic and and feeble, was taken sick on the 18th of January, 1850. Pain, metastatic, but confined chiefly to the upper joints. She was purged freely with croton oil every day, until the 21st, when I prescribed the following. ℞. Mass. Hydrarg. gr. xx. Quiniæ Sulph. ʒss.; M. Pil. 6. S. One pill every two hours. On the 24th purged her again with croton oil, and discharged her cured on the 26th.

Case 12.—Mrs. Fanny ——, aged 35, had been suffering from dyspepsia for more than a year. About ten days before I saw her, which was on the 18th of March, 1850, she felt considerable pain in the shoulders, back of the neck, and sides of the chest. Her bowels were confined, and she had some febrile movement. I gave her alternately, every day, a drop of croton oil, and fifteen grains of quinine, for four days. She then took ten grains of Dover's powder every night, followed by a drop of croton oil in the morning, until the 24th of March, when she was discharged, cured.

*Remarks.*—A celebrated physician being once asked what he considered the most successful treatment of acute rheumatism, answered, "six weeks"—thereby plainly intimating his opinion of the intractable nature of the disease. While I am ready to admit that there is *some* truth in this opinion, and that rheumatism now and then assumes a form which might well entitle it to a place among the *opprobria medicorum,* I am not at all prepared to entertain the notion, that early and judicious treatment has no power, in a majority of cases, to alleviate its pangs, or shorten its duration. On the contrary, I believe that rheumatism is as amenable to proper management as any disease of equal severity.

Within the past five years, I have accurately noted the history and treatment of about *thirty* cases of acute articular rheumatism, from among which the twelve cases just detailed are indiscriminately taken. In these, it will be seen, the average duration of the disease, was 5½ days; the longest being 14, and the shortest 2 days. *Croton oil* was used in 11, *colchicum* in 6, *quinine* in 6, *bloodletting* in 2, and *iodide of potassium* in 2. I desire to say a word or two upon each of these agents.

Of *croton oil* as a purgative in acute rheumatism, I am prepared to speak in the highest terms. Cathartics have always stood in the front rank of remedies in this disease, but I am disposed to believe that the efficacy of croton oil does not depend entirely upon its cathartic properties; it possesses a power over the disease beyond these, and apparently not dependent upon them, for other cathartics, which act as powerfully and as promptly, producing similar watery stools, do not bring a like amount of relief to the patient. I do not say that it is a *specific*, for I am not a believer in the doctrine of specifics, in medicine; that doctrine has put more stumbling blocks in the way of medical progress than all the open quackery of the past half century. I merely desire to state, that after a fair trial, in a number of cases accurately observed, where there was scarcely a possibility of falling into error, I believe that the croton oil is the *best* single remedy in the treatment of acute rheumatism; and I am thoroughly convinced, that it is as justly entitled to the term *specific*, in this disease, as is quinine in miasmatic fever. I speak now, not of chronic rheumatism, nor of that which results from a syphilitic taint, but of the acute inflammatory affection. In all the eleven cases, in which it was given, its beneficial effect was palpably evident, but particularly as in cases 5, 6, 8, 10, 11, and 12.

Dr. Wm. A. Thom, an intelligent physician of Northampton County, to whom I am indebted for the first hint in regard to the value of croton oil in this disease, informed me, about two years ago, that he had used it in several severe cases, and uniformly with success.

*Colchicum* has uniformly disappointed me. In cases 1, 4, 7, it was fairly tried, and although it invariably acted freely upon the bowels, there was no alleviation of the distressing symptoms. In the chronic disease, I think I have sometimes derived great benefit from it, but in the acute form, *never*. It may be, that I have been so unfortunate as always to get hold of an inferior preparation, but some how or other, I have taken the notion, that when a patient is treated with colchicum *only*, he would do just as well to take old Dr. Warren's *six weeks* instead.

*Quinine* I would place next to croton oil in the treatment of rheumatism. That it is decidedly *sedative* in large doses, is undeniable, and therefore it may be exhibited during the febrile stage. Given after the use of the lancet, in highly inflammatory cases, or after powerful purgation with croton oil, it has been productive of the happiest effects in my hands. I never give to an adult a smaller dose than ten grains, repeated three or four times a day; less than this would be worse than useless. It is particularly serviceable in those cases which perspire most profusely.

It seems to me that *bloodletting* possesses no direct *curative* power in the treatment of rheumatism. In robust persons, with high fever, intense pain, and a full, bounding pulse, I think it subserves a useful purpose in rapidly subduing the over-excited circulation, thus placing the system in a better condition to respond to the impression of true curative agents. It is true that bloodletting is one of the most powerful antiphlogistics, and that rheumatism, in its acute form, presents undeniable evidences of its highly inflammatory character, nevertheless it has long since been voted an inflammation, *sui generis*, and I believe there is a great deal too much of the *neuralgie* about it to justify the heroic use of the lancet so warmly advocated by Bouillaud. *Topical* depletion has never been of the least service in my hands, except where the disease was confined to a single joint, *with no disposition toward metastasis.*

When the disease assumes the chronic form, or if there should exist a scrofulous, or syphilitic taint, no remedy will be found equal to the *iodide of potassium.* I usually order five grains four times a day, to be taken in hop tea, the bowels in the meantime being opened every day with the black draught, or other cathartic. I have rarely seen the most obstinate cases refuse to yield to this treatment.

In the second attack of Case 7, almost immediate relief followed copious purgation with the decoction of *may-apple* (podophyllum,) but it is very unphilosophical, particularly in so uncertain a science as Therapeutics, to deduce a general conclusion from a solitary observation, so I will not venture to recommend the may-apple until authorized by further trial. I have adverted to it, merely because it forms a part of the history of the case; and with the remark, that it is considered by all of the old *negro-doctors* in this section of country as a *specific* in rheumatism, I dismiss the subject.

*Norfolk, Va., Sept. 5th,* 1850.

